# Investigation of heterocellular features of the mouse retinal neurovascular unit by 3D electron microscopy

**DOI:** 10.1111/joa.13721

**Published:** 2022-07-16

**Authors:** Mona J. Albargothy, Nadhira N. Azizah, Sarah L. Stewart, Evan P. Troendle, David H. W. Steel, Tim M. Curtis, Michael J. Taggart

**Affiliations:** ^1^ Biosciences Institute Newcastle University Newcastle upon Tyne UK; ^2^ Wellcome Wolfson Institute for Experimental Medicine Queen's University Belfast Belfast UK

**Keywords:** astrocytes, basement membrane, capillaries, endothelium, macroglia, neurons, Neurovascular Unit, Peg‐and‐socket, pericytes, retina, SBF‐SEM

## Abstract

The retina has a complex structure with a diverse collection of component cells that work together to facilitate vision. The retinal capillaries supplying the nutritional requirements to the inner retina have an intricate system of neural, glial and vascular elements that interconnect to form the neurovascular unit (NVU). The retina has no autonomic nervous system and so relies on the NVU as an interdependent, physical and functional unit to alter blood flow appropriately to changes in the physiological environment. The importance of this is demonstrated by alterations in NVU function being apparent in the blinding disease diabetic retinopathy and other diseases of the retina. It is, therefore, imperative to understand the anatomy of the components of the NVU that underlie its functioning and in particular the nanoscale arrangements of its heterocellular components. However, information on this in three spatial dimensions is limited. In the present study, we utilised the technique of serial block‐face scanning electron microscopy (SBF‐SEM), and computational image reconstruction, to enable the first three‐dimensional ultrastructural analysis of the NVU in mouse retinal capillaries. Mouse isolated retina was prepared for SBF‐SEM and up to 150 serial scanning electron microscopy images (covering *z*‐axes distances of 12–8 mm) of individual capillaries in the superficial plexus and NVU cellular components digitally aligned. Examination of the data in the *x*‐, *y*‐ and *z*‐planes was performed with the use of semi‐automated computational image analysis tools including segmentation, 3D image reconstruction and quantitation of cell proximities. A prominent feature of the capillary arrangements in 3D was the extensive sheath‐like coverage by singular pericytes. They appeared in close register to the basement membrane with which they interwove in a complex mesh‐like appearance. Breaks in the basement membrane appeared to facilitate pericyte interactions with other NVU cell types. There were frequent, close (<10 nm) pericyte–endothelial interactions with direct contact points and peg‐and‐socket‐like morphology. Macroglia typically intervened between neurons and capillary structures; however, regions were identified where neurons came into closer contact with the basement membrane. A software‐generated analysis to assess the morphology of the different cellular components of the NVU, including quantifications of convexity, sphericity and cell‐to‐cell closeness, has enabled preliminary semi‐quantitative characterisation of cell arrangements with neighbouring structures. This study presents new data on the nanoscale spatial characteristics of components of the murine retinal NVU in 3D that has implications for our understanding of structural integrity (e.g. pericyte‐endothelial cell anchoring) and function (e.g. possible paracrine communication between macroglia and pericytes). It also serves as a platform to inform future studies examining changes in NVU characteristics with different biological and disease circumstances. All raw and processed image data have been deposited for public viewing.

## Graphical abstract

In the present study, we provide a three‐dimensional ultrastructural analysis of the mouse retinal neurovascular unit (NVU). Pericytes were found to interweave in a sheath‐like fashion with the basement membrane while making regular peg‐and‐socket‐like interactions with capillary endothelial cells. Convoluted macroglial homo‐ and heterocellular (with neurones) interweaving along the capillary length was observed. On occasions macroglial and neuronal cells, respectively, came in close proximity to pericytes and the basement membrane. The complex nanoscale spatial characteristics of the NVU revealed herein will inform future studies examining changes in NVU structure and function with different biological and disease circumstances.

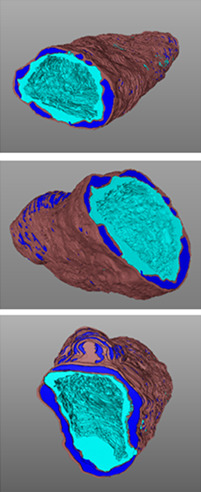

## 1 INTRODUCTION

The retina is a multi‐layered structure with a diverse collection of component cells that work together to produce a complex visual output (Hoon et al., 2014). Light is detected by rod and cone photoreceptors which convert it into an electrical signal for propagation through the retinal circuitry to the optic nerve and subsequently the visual cortex of the brain. It is well known that the retina is one of the most metabolically active tissues in the body, with a high demand for oxygen and nutrient delivery from the vasculature (Morrow, 2014). To fulfil these needs, the retina is nourished by two distinct vascular beds: the intraretinal vasculature that supplies the inner retina and the choroidal vessels which serve the outer retina (Flammer & Mozaffarieh, 2008).

The blood vessels of the inner retina form part of the retinal neurovascular unit (NVU). The NVU refers to the physical and functional relationship among the capillary blood‐vessel endothelial cells, pericytes, macroglia (Müller glia and astrocytes), microglia and neurons (ganglion cells, bipolar cells, horizontal cells and amacrine cells) of the retina (Simó et al., 2014). Collectively, the cells of the NVU play a key role in the maintenance of the inner blood retinal barrier, in mediating cell‐to‐cell survival via paracrine signalling, and in matching blood flow to the metabolic needs of the retina through a process known as neurovascular coupling (Duh et al., 2017; Metea & Newman, 2007). The latter is particularly important given the absence of autonomic innervation in the retina (Ye et al., 1990). Disruption of the integrity of the retinal NVU has been implicated in the pathogenesis of several retinal diseases, including diabetic retinopathy, glaucoma and retinal neurodegenerative disorders (Ivanova et al., 2019; Lechner et al., 2017; Weinreb et al., 2014). Despite this, the three‐dimensional ultrastructure of the retinal NVU remains to be fully characterised and quantitative methods for describing its key features have yet to be developed.

The retinal NVU in mice and humans has been previously analysed in two dimensions at the ultrastructural level using conventional transmission electron microscopy (TEM) (Fehér et al., 2017; van der Wijk et al., 2018). While this technique has provided valuable information on its structure, a greater understanding of the anatomical arrangements of the different cellular components, and their possible heterocellular interactions, requires analysis in three dimensions with nanometre spatial resolution. Serial block‐face scanning electron microscopy (SBF‐SEM), a technique developed by Denk and Horstmann (2004), allows for the automatic serial thin sectioning and scanning of an embedded tissue or sample in a scanning electron microscope. This allows for the collection of multiple serially registered ultrastructural images which, together with computational image reconstruction software, can provide a three‐dimensional view of tissue microanatomy, with resolutions as low as 3–5 nm (Cocks et al., 2018; Nian et al., 2021). In the retina, the use of SBF‐SEM has so far been limited to studies focused on synaptic connectivity mapping (Behrens et al., 2021; Graydon et al., 2018; Helmstaedter et al., 2013; Wool et al., 2019), the packing geometry of retinal pigment epithelial cell granules (Agrawal et al., 2017) and neuronal changes associated with retinal degenerative phenotypes (Agrawal et al., 2017; Kerov et al., 2018).

In the present work, we have investigated for the first time the murine retinal NVU using SBF‐SEM to define its ultrastructural characteristics in three dimensions. Image analysis tools have also been developed to quantitatively describe key aspects of the mouse retinal NVU, providing a basis in the future to better understand how this structure is altered during retinal disease.

## 2 METHODS

### 2.1 Sample collection and preparation

Tissue samples used in this study were from healthy mouse retina and all capillaries sampled were in the superficial vascular plexus. An adult C57BL/6 mouse (3 months) was killed by Schedule 1 procedure according to the Animals Scientific Procedures Act (ASPA) 1986, the eyes extracted and retinas micro‐dissected into fixative (2% glutaraldehyde in 0.1 M sodium cacodylate buffer, pH 7.3) for a minimum of 12 h for electron microscopy. The samples were then processed using a heavy metal staining protocol to provide for an electron dense surface for the beam to interact with (Cocks et al., 2018; Wilke et al., 2013).

The samples were embedded into resin (Taab 812 epoxy resin) and left to polymerise for a minimum of 36 h. The resin blocks were trimmed using a razor blade to form a trapezoid block face. The blocks were then trimmed to approximately 0.75 mm × 0.75 mm and glued onto a pin.

### 2.2 SBF‐SEM settings—Image collection

Resin‐embedded retinal tissue samples were imaged using a Zeiss Sigma SEM chamber (Zeiss) combined with Gatan 3View software (Gatan). SBF‐SEM collects multiple serial‐registered digitised images, following automated ultramicrotome sectioning of the block face in situ. The Gatan 3View software was used to identify retinal capillaries of interest and to ensure that they remained in the centre of the field of view as the images were collected. The diamond ultramicrotome was set to cut sections at either 100 or 120 nm and 100–150 consecutive micrographs were captured for each capillary covering a z distance of 12–18 μm. Image dimensions were set to 3000 × 3000 pixels, at a pixel size of 6 nm, with a 20 μs/pixel dwell time. All raw data analysed in the report are available at the following dois: 10.25405/data.ncl.18545270 (capillary 1); 10.25405/data.ncl.18550982 (capillary 2); 10.25405/data.ncl.18551036 (capillary 3); 10.25405/data.ncl.18550856 (capillary 4).

### 2.3 Image processing

**FIGURE 1:**
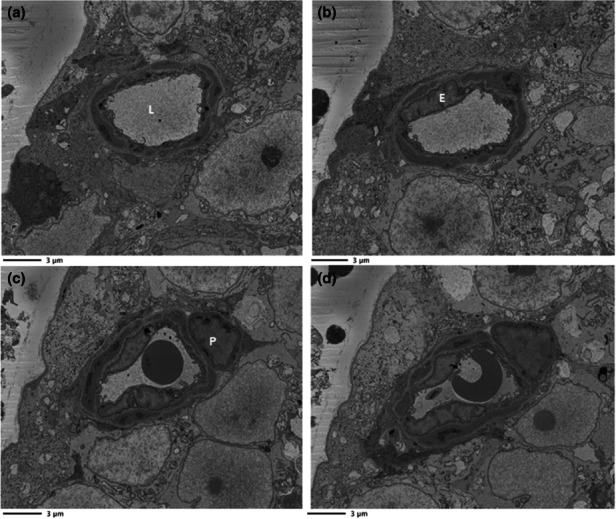
Raw dataset for mouse retinal capillary. A collection of SBF‐SEM micrographs taken from a mouse retinal capillary to display the morphological changes along the capillary length. (a) Slice 1 (0.12 μm), (b) slice 50 (6 μm), (c) slice 100 (12 μm) and (d) slice 120 (14.4 μm), Lumen: L, Pericytes: P, Endothelium: E.

DM3 files collected from the SBF‐SEM were opened in Microscopy Image Browser (MIB v2.1) for post‐processing (http://mib.helsinki.fi/) (Belevich et al., 2016). DM3 image files opened in MIB are displayed with low contrast, so cellular features cannot be easily distinguished. Image contrast was therefore optimised, and contrast normalisation was performed throughout the *z*‐dimension for all images within the stack. Images were then aligned using the drift correction tool. To reduce the dataset size, and speed up dataset operations, the images were converted from 16‐ to 8‐bit, and the processed dataset was saved as an Amira mesh binary (.am) file. An example of individual microscopic images obtained from a stack of digitally aligned image data are given in Figure 1. Video files of the image stacks for each capillary are provided in Files S1–S4 (10.25405/data.ncl.19086755; 10.25405/data.ncl.19086752; 10.25405/data.ncl.19086617; 10.25405/data.ncl.19086746).

Before segmentation, the cellular components of the NVU were inspected and assigned an identity based on either their location in the capillary or their morphology. The endothelium lines the lumen of the capillary and is thus the innermost layer of the vasculature. As demonstrated in Figure 1, the endothelium is surrounded by pericytes, which appear to have a spindle‐like morphology. The basement membrane (BM) has a lighter tone and surrounds the pericytes separating them from the endothelium. Macroglia were identified by their abundance of electron‐dense endoplasmic reticulum in the cytoplasm (Bianchi et al., 2015; Wakakura & Foulds, 1988). Digital zooming of images was used to identify juxtaposed cell boundaries from two neighbouring cells (File S5: 10.25405/data.ncl.19597255). For the purposes of this study, Müller cells and astrocytes were grouped together as macroglia since in most cases they could not be discriminated from one another in SBF‐SEM images. The only exception was in one image stack, where an astrocyte was clearly identified based on the presence of its nucleus within the field of view (File S6: 10.25405/data.ncl.19597261). Our imaging was restricted from the inner limiting membrane (ILM) to the ganglion cell layer (GCL) and it is recognised that it is only astrocytes, and not the Müller cells, that have their cell bodies situated in this region of the retina (Reichenbach & Bringmann, 2019). Retinal ganglion cells (RGCs) were identified by their electron‐lucent appearance, their smooth cytoplasm which lacked reticulation and the fact that their nuclei were visible, appearing rounded with indentations.

### 2.4 Image segmentation and 3D reconstruction

Each feature of interest of the NVU was segmented (delineated by colour‐coding) throughout z‐stacks of data using MIB (http://mib.helsinki.fi/). Due to the complexity of the cellular morphology, a combination of semi‐automatic interpolation and manual image segmentation was performed with the use of tools, filters and interpolation in XY, XZ or YZ planes. Manual segmentation was performed by selecting the MIB brush tool to trace a cell membrane or feature which was then filled (using F or shift + F for the whole dataset). The selection was added to a material (A or shift + A) depending on the cell type. Colour‐coded area lists corresponding to specific features in the images were assigned as follows: the BM (brown), endothelial cell (aqua), pericyte (blue), macroglia (red and purple shades) and neurons (green shades).

To speed up segmentation, interpolation was performed. Structures with a similar morphology throughout the slices, often including endothelial cells and neurons, were manually segmented every two to four sections, with gaps automatically segmented using MIB's interpolation tool. Any segments which did not outline the cellular membrane or feature accurately were manually corrected and retracted using the brush tool.

After segmentation was complete, the file was exported to Amira mesh binary format (.am). Complete segmented objects were then imported to Amira software (ThermoFisher, http://www.fei.com/software/amira‐3d‐for‐life‐sciences/) or ARIVIS, Vision 4D (https://imaging.arivis.com/en/imaging‐science/arivis‐vision4d) for further visualisation and rendering in 3D.

### 2.5 Identification of Peg‐and‐socket formations

Inspection of the data revealed that pericyte‐endothelial membrane convolutions of a peg‐and‐socket‐like formation were a common feature of the retinal NVU (detailed in Results and Discussion). A quantitative analysis to calculate the number of such pericyte‐endothelial arrangements was performed by manually counting these structures. Each dataset was analysed on a slice‐by‐slice basis along the pericyte‐endothelial borders for the presence of peg‐and‐sockets. By examining pericyte‐endothelial cell boundaries in four retinal capillaries, we determined several compulsory criteria to define peg‐and‐sockets, which can be either pericyte or endothelial facing. These features must:

1. Span across a minimum distance of 200 nm in the z‐dimension.
2. Contain at least one section where the peg attaches to the cell body
3. Transverse the BM.
4. Be engulfed by a neighbouring cell.
5. Have a minimum transverse extent (width) of 0.07 μm and a minimum longitudinal extent (length) of 0.1 μm.

A quantitative image analysis pipeline created in ARIVIS was used to assess the validity of the manual identification of peg‐and‐sockets. This pipeline first morphologically dilated each endothelial object and pericyte‐endothelial intersects were detected by the software programme (examples of such are displayed in green in File S7: 10.25405/data.ncl.19597264). Then, pixels that are shared between the dilated endothelium and the adjacent pericytes (i.e. the intersection of these two materials) are labelled as potential pericyte‐endothelial interaction sites. This analysis tagged all pericyte‐endothelial peg‐and‐socket features identified manually as well as regions where the two materials were sufficiently close, but not in peg‐and‐socket formations (i.e, they did not fit the criteria listed above).

### 2.6 Proximity analysis

In plane minimal distances between the boundaries of all pairwise combinations of intercellular and cellular‐BM features were used to analyse proximity using MATLAB r2021b with Image Processing Toolbox™ (MATLAB; https://www.mathworks.com/products/matlab.html). For each image slice and feature pair, two minimal distance histograms were generated; one contained the minimal distances from each boundary pixel of the first feature in the pair to the nearest boundary pixel of the second, and the other contained the minimal distances in the opposite direction (i.e. from each boundary pixel in the second feature of the pair to the nearest boundary pixel of the first). The proximity histograms for each feature pair were consolidated into a mean proximity histogram using all data in the image stack. Cumulative distribution functions were calculated for each mean proximity histogram, which assesses the total percentage of boundary pixels that lie within a given distance between each feature pair (i.e. if the y‐value of the cumulative distribution function chart at 50 nm is 5%, 5% of all boundary pixels comprising the first feature are within 50 nm of the second). The MATLAB proximity analysis script may be found in File S8 (10.25405/data.ncl.19087070).

### 2.7 Morphological analysis

Several image analysis procedures were implemented in MATLAB to quantify the morphological properties of the NVU features of interest. The area (A) of each object was determined by counting the number of pixels comprising it. The perimeter (P) of each object was measured by first using MATLAB to determine boundary pixels, and then estimating the perimeter by analysis of its Freeman chain code (Freeman, 1961). Convex hull areas (CA) and perimeters (CP), which enclose the smallest convex area about each object, were calculated equivalently. Radii were measured by determining the central coordinate of the subject area and calculating the 2D Euclidean distance (i.e. $r=\sqrt{∆x^{2}+∆y^{2}}$) from it to each of the pixels composing the boundary, taking the mean of this distribution.

Ratios of these key metrics, which are standard in morphological image analysis (Mingqiang et al., 2008) were also recorded for interpretation. Convexity (CP:P) denotes the degree that the perimeter of an object deviates from that of its convex hull. Solidity (A:CA) denotes the degree to which an object's area deviates from that of its convex hull. Sphericity denotes the degree to which an object approaches a perfect circle (i.e. Rmin:Rmax from the distribution of boundary‐centroid distances used to determine R).

A pictographic summary of the various morphological analyses undertaken in this study is provided in File S9 (10.25405/data.ncl.19087076). The MATLAB morphological analysis script may be found in File S10 (10.25405/data.ncl.19087079).

## 3 RESULTS AND DISCUSSION

### 3.1 Ultrastructural features of the capillaries

Four capillaries from an adult mouse retina underwent serial imaging with SBF‐SEM. An example of the cellular arrangements of the NVU obtained by SBF‐SEM is given in Figure 1. Changes in cell shape and positioning were evident throughout the z‐stacks of data.

**FIGURE 2:**
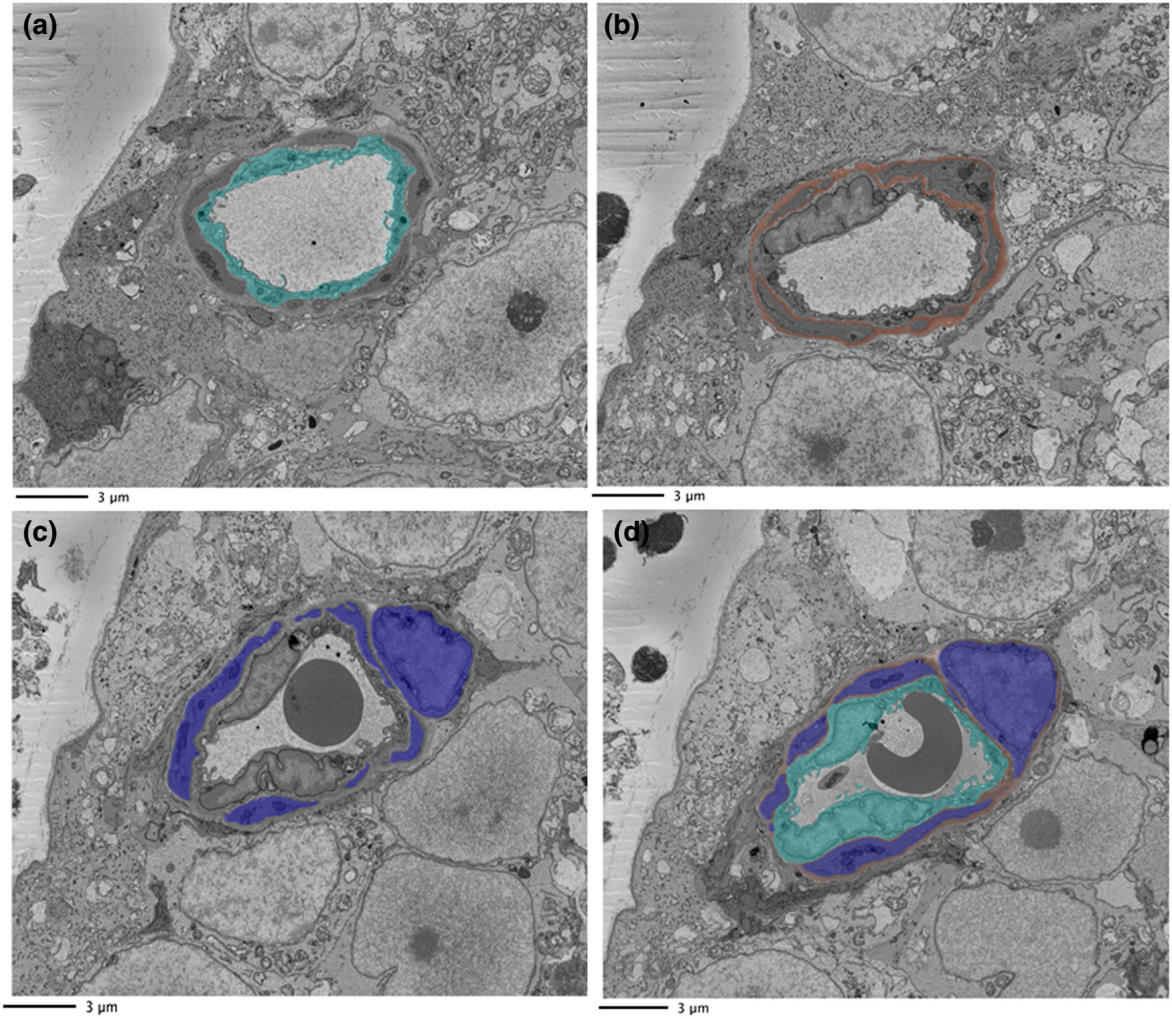
Segmented vasculature. The vasculature was identified, and each component was assigned a colour for segmentation through the data stack and subsequent visualisation in 3D reconstructions. (a) endothelium (aqua); (b) basement membrane (brown); (c) pericytes (blue); (d) complete vasculature.

**FIGURE 3:**
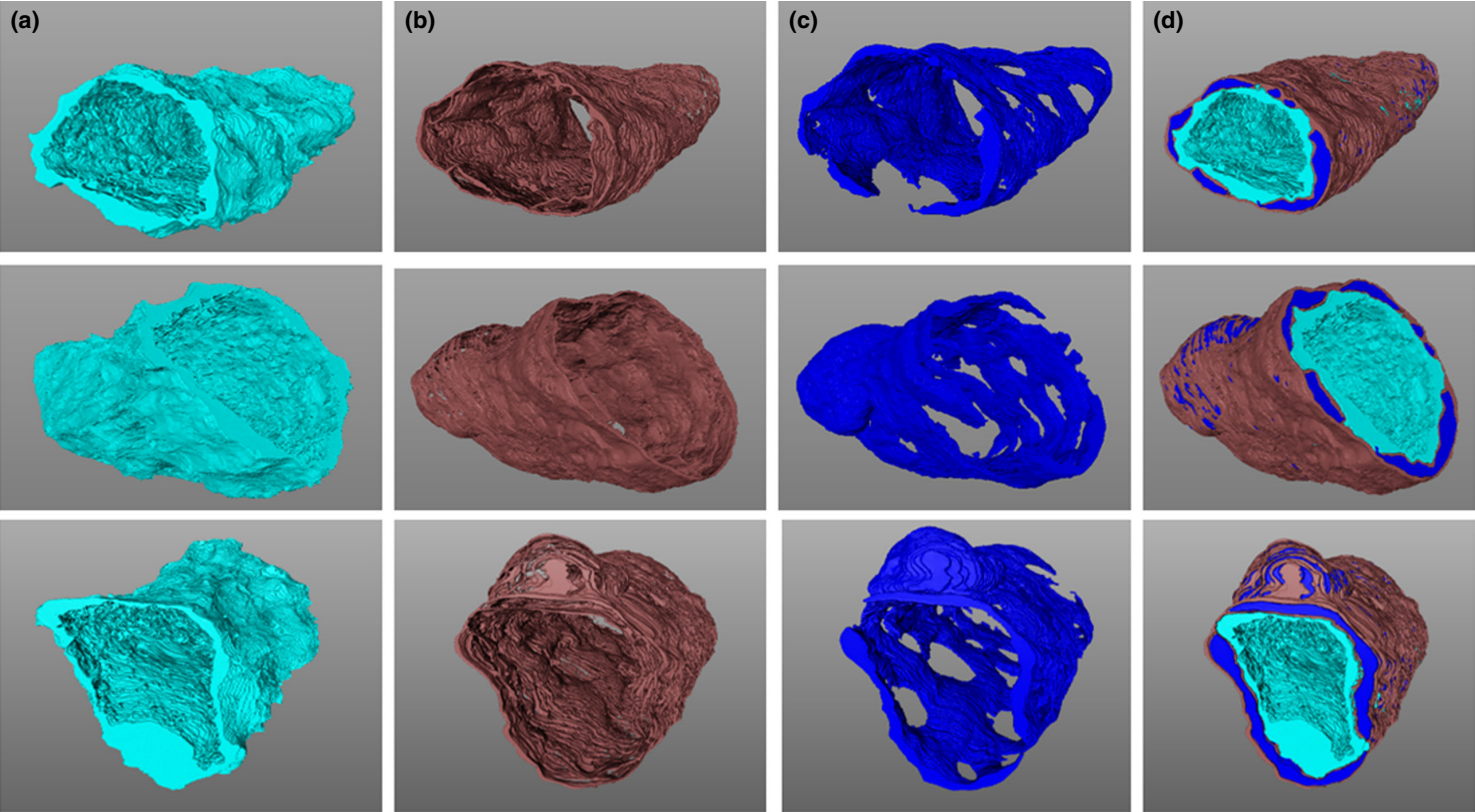
3D reconstruction of mouse capillary vasculature. Displayed are features associated with a 3D reconstruction of the vasculature of capillary 1. Column a: endothelium (aqua); column b: basement membrane (brown); column c: pericytes (blue); and column d: combined vasculature.

An example of segmentation of capillary features is provided in Figure 2. Indicated in four panels is the appearance of the endothelium (aqua), BM (brown) and pericyte (blue) within the same image section. The meshwork appearance of the BM interweaving with the pericyte, itself forming a convoluted appearance around the vessel, is evident here and in the 3D digital reconstructions of a complete data stack (Figure 3; see also video files of stacks of segmented capillaries in Files S11–S13: 10.25405/data.ncl.19086398; 10.25405/data.ncl.19086401; 10.25405/data.ncl.19086131).

As the BM both enveloped and interwove with the pericyte this influences the closeness of pericytes with underlying endothelium. Several TEM studies have attempted to report the relationship of pericytes to endothelial cells in retinal capillaries. Estimates from examination of singular ultra‐thin sliced images in the *x*‐ and *y*‐axes suggest that pericyte coverage of the endothelial surface ranges between 41% and 58% in rodent retinal capillaries (Frank et al., 1987; Tilton et al., 1985). Using our proximity analysis on capillary 1 (see “Methods” section), we have quantified pericyte ensheathment of the endothelium along the *z*‐axis, with a stack mean and standard deviation of 35.3 ± 23.7% of the abluminal endothelial surface lying within 200 nm of the pericyte layer (range 12%–100%).

### 3.2 Pericyte and endothelial connections

**FIGURE 4:**
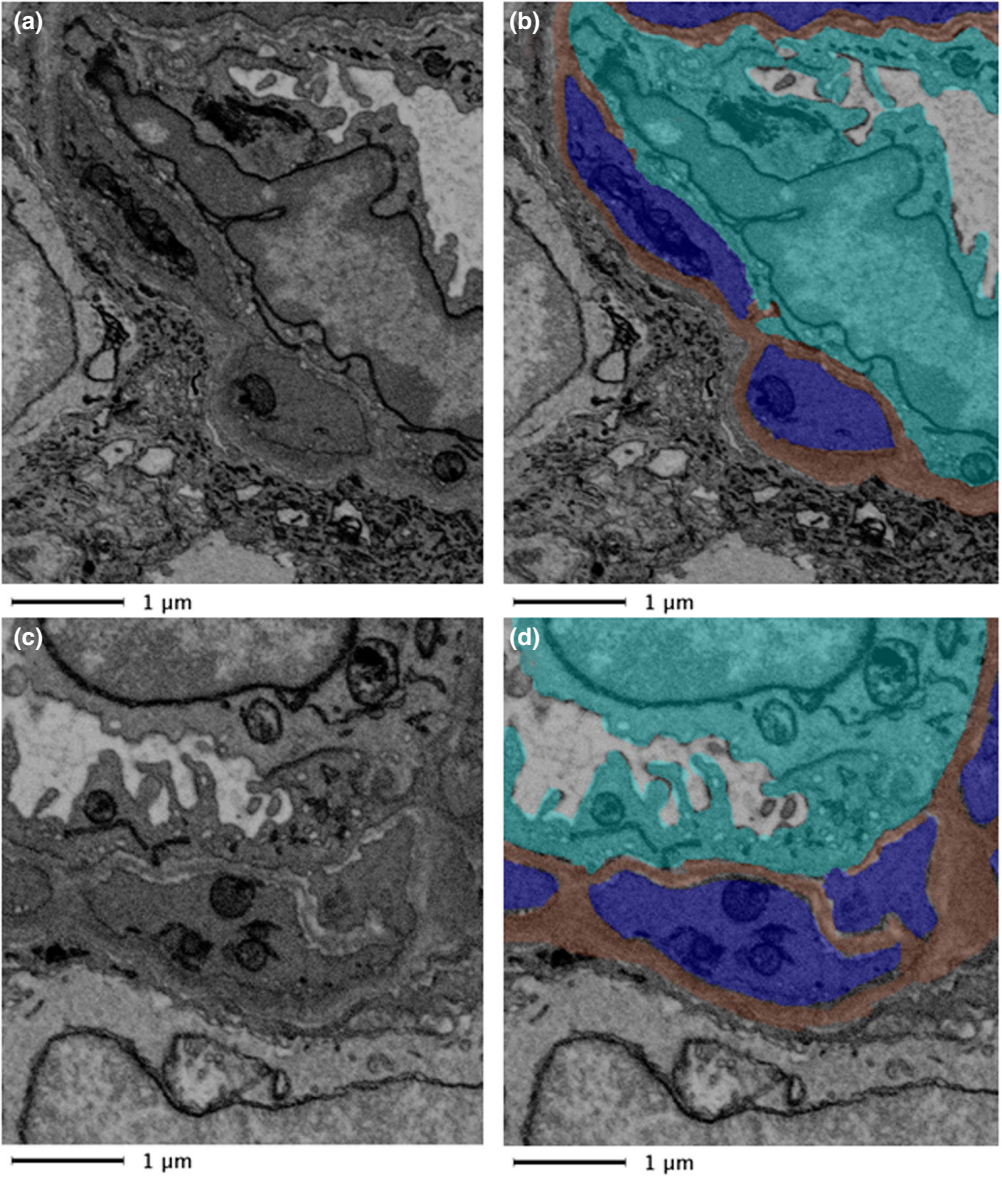
Pericyte‐endothelial interaction via direct contact. Close contacts of a pericyte (blue) and endothelial cell (aqua) in absence of a bordering basement membrane (brown) are indicated for the raw data (a and c) and segmented data (b and d) of two sections 1.2 μm apart.

**FIGURE 5:**
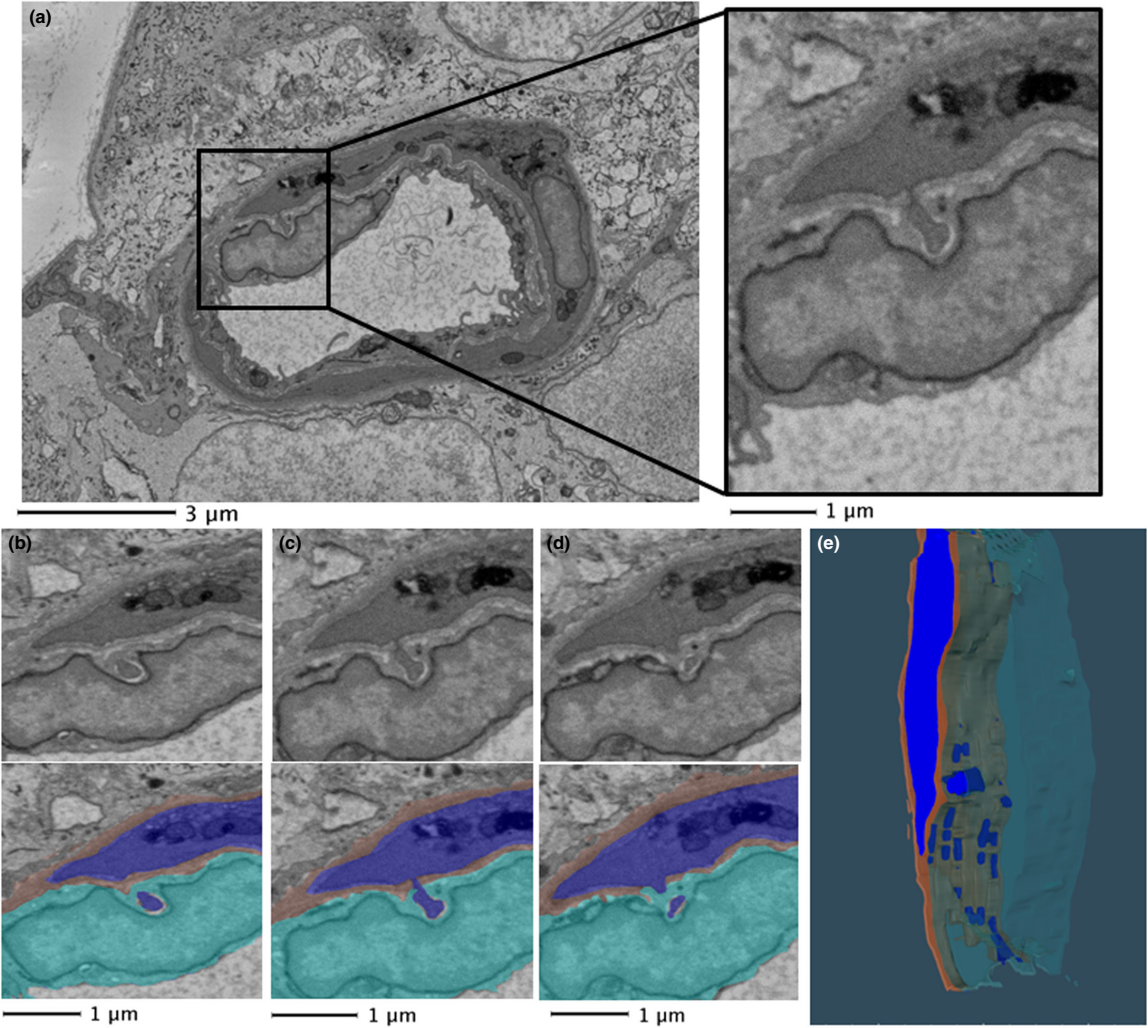
Peg‐and‐socket pericyte‐endothelial formation spanning across multiple sections. (a) Peg‐and‐socket formation identified on a capillary (magnified area evident on right hand side bounded by black box). (b–d) This peg and socket spanned across three sections (minimum 0.360 μm). (e) Displays the 3D nature of peg and sockets; corresponding video file can be found in File S15 (10.25405/data.ncl.19086197). Endothelium: aqua; basement membrane: brown; Pericytes: blue.

The BM wraps around the abluminal endothelium and pericytes for the most part forming a barrier separating the two cells. However, as indicated above, along the capillary length gaps in the BM were observed allowing pericytes and endothelial cells to come into closer contact. These interactions were of two types, namely, direct contact points (Figure 4) and peg‐and‐socket type contact areas (Figure 5; File S14: 10.25405/data.ncl.19087082).

**FIGURE 6:**
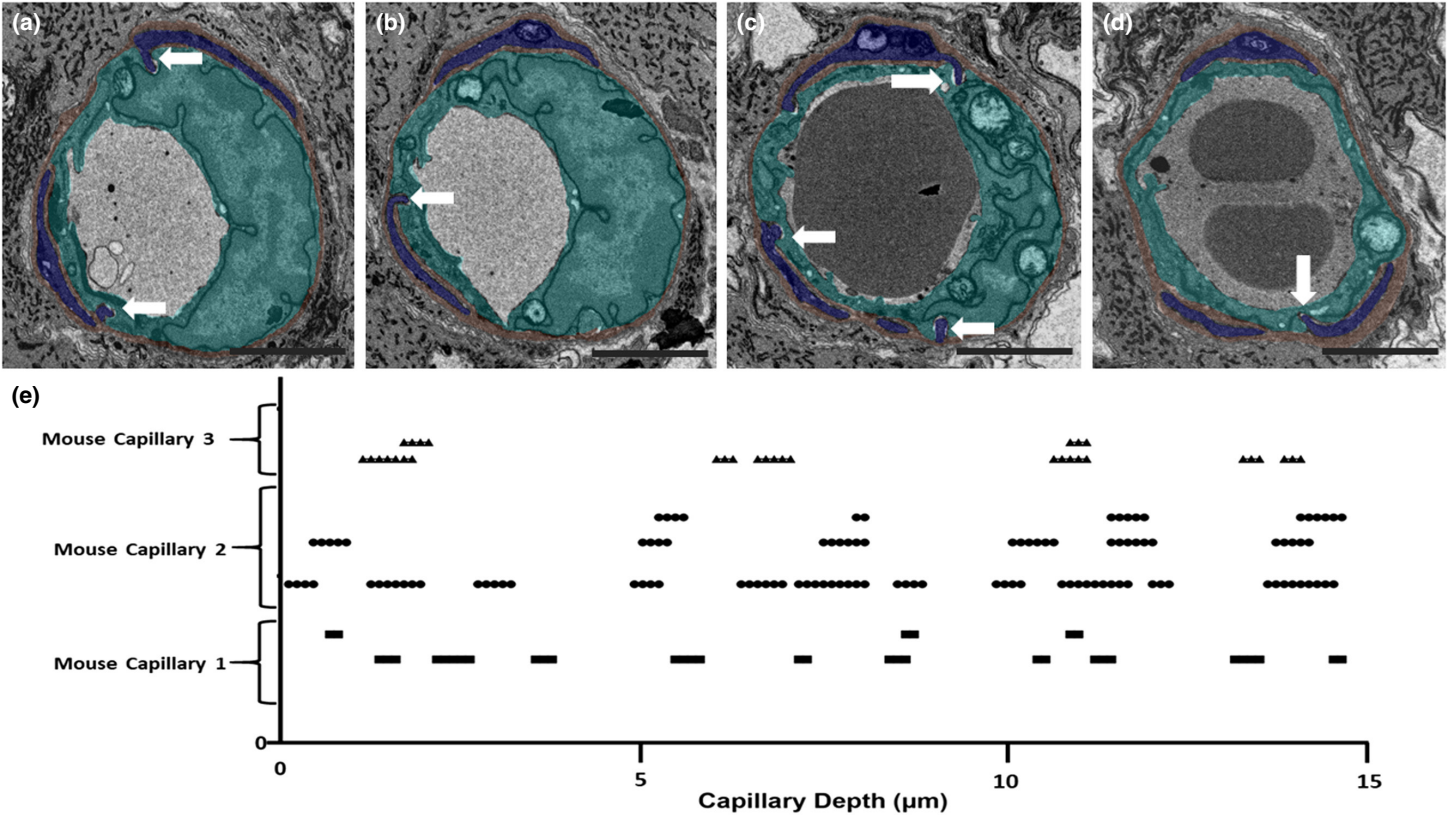
Distribution of peg and sockets across mouse capillaries. The distribution of peg‐and sockets evident across 150 sections examined in three capillaries. Note more than one peg‐and‐socket may appear in the same sections. (a) Two peg‐and‐sockets (indicated by white arrows) from mouse capillary 2 on slice 44 at depth 4.4 μm. (b) Additional peg from mouse capillary 2 on slice 61 at 6.1 μm depth. (c) Three peg‐and‐sockets from mouse capillary 2 on slice 89 at depth 8.9 μm. (d) A single peg‐and‐socket from mouse capillary 2 on slice 143 at depth 14.3 μm. (e) Peg‐and‐socket distribution graph for mouse capillaries 1, 2 and 3. Endothelium: aqua; basement membrane: brown; pericytes: blue. Scale bar: 3 μm.

Such features have been reported from 2D TEM studies of Toussaint & Dustin, 1963 and Carlson (1989). SBF‐SEM imaging enables better visualisation of these features in 3D and our data reveals a characteristic shape of the peg‐and‐socket abutments of pericyte and endothelial cell membranes as displayed in more detail in Figures 5 and 6 (see also Files S15 and S16: 10.25405/data.ncl.19086197; 10.25405/data.ncl.19086335). The pericyte or endothelial cell membrane was observed to protrude towards, and be enveloped by, the neighbouring cell plasma membrane although pericyte protrusion was more common.

Pericyte‐endothelial peg‐and‐socket formations were noted to occur at several points throughout the data stacks of each capillary analysed. Over the depth of 15 μm in each of the capillaries, peg‐and‐socket formations appeared many times with each formation spanning across several sections. Their distributions are shown in Figure 6. This suggests the nature of pericyte–endothelial interactions commonly occur in peg‐and‐socket‐like formations. Moreover, these heterocellular connections are likely to provide structural anchoring between the two cell types. As such, these structures may be important for maintaining the structural integrity of the inner blood‐retinal‐barrier. More generally, the pericytes and endothelium lying on either side of a shared BM, and encircling the capillary lumen along the *z*‐axis, support a role in the integrated regulation of vascular diameter and thereby retinal blood flow and nutrient exchange (Geevarghese & Herman, 2014).

It is of interest that pericyte‐endothelial cell relationships can take on several formations depending on the tissue/organ served by the capillary, for example, close alignment/contact of pericytes and endothelial cells in placenta, pericyte pegs approaching endothelial cells in the kidney and peg‐and‐socket arrangements in the brain, in addition to the retina (Harris et al., 2021; Ornelas et al., 2021; Stefanska et al., 2016).

### 3.3 Macroglial ensheathment of retinal capillaries

**FIGURE 7:**
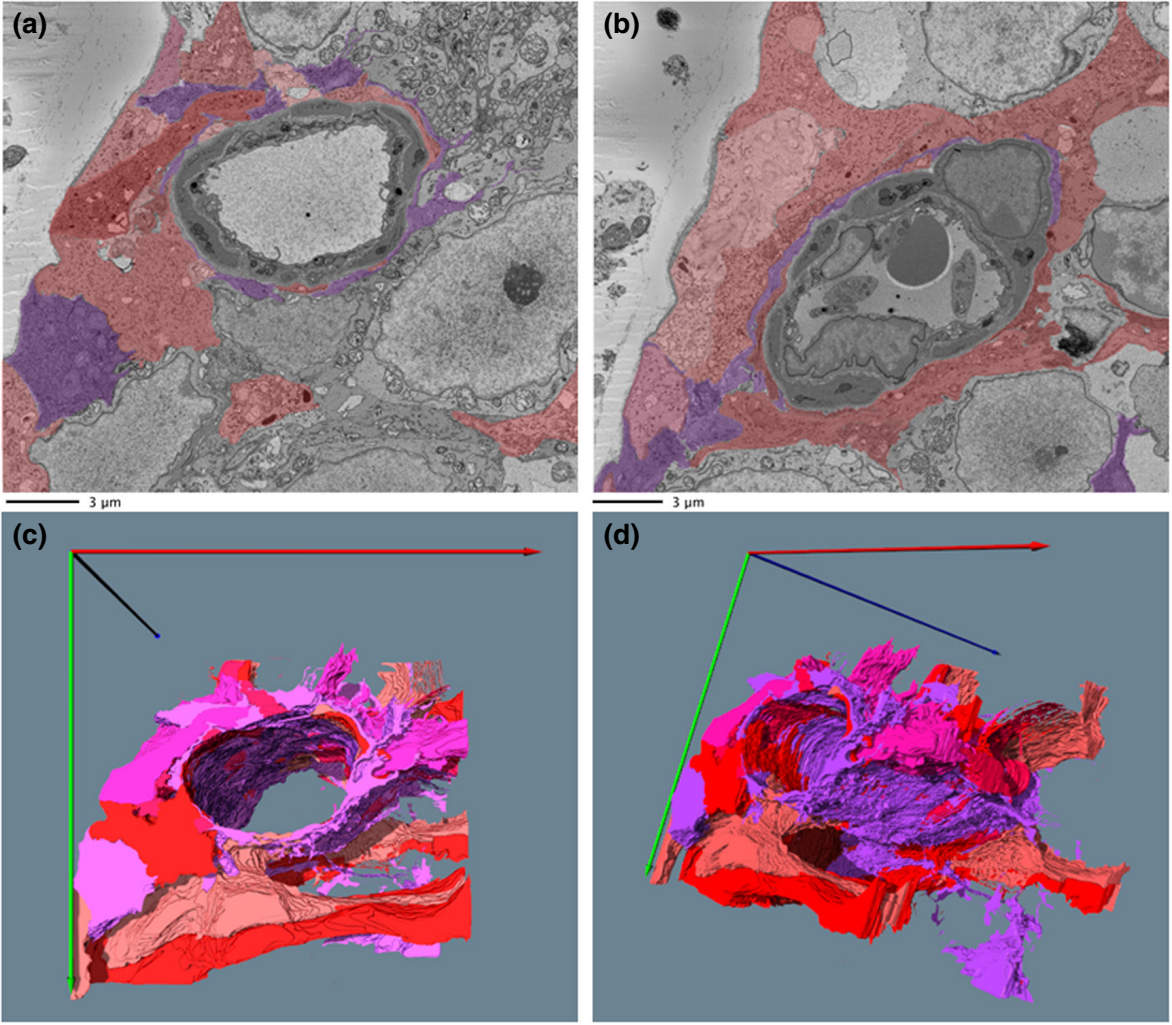
Segmented data and 3D reconstruction of macroglia surrounding the vasculature. (a, b) Macroglia in (red and purple shades) can be seen to wrap around the vasculature across different sections along capillary depth. Purple shading depicts a cell identified as an astrocyte. Displayed in a and b are sections 6 μm apart. 3D reconstruction views (c, d) show the complexity of the wrapping along 18 of vessel (the corresponding video file can be found in File S17 (10.25405/data.ncl.19086341).

Previous TEM studies have suggested that the macroglia, especially Müller cells, come into close contact with the outer vascular BM of the retinal pericytes. We frequently observed this through examination of the SBF‐SEM datasets, while revealing the intricacy and complexity of such interactions in 3D. The interweaving of the macroglia is complex and extensive along the capillary length (Figure 7 and File S17: 10.25405/data.ncl.19086341). The macroglia forms an interlocking, ‘patchwork‐ quilt’ style pattern as they lie over the outer vascular BM. Macroglia cell processes are also observed to extend in directions distant from the capillary and interacting with other cellular (neuronal) components of the retina (see below).

**FIGURE 8:**
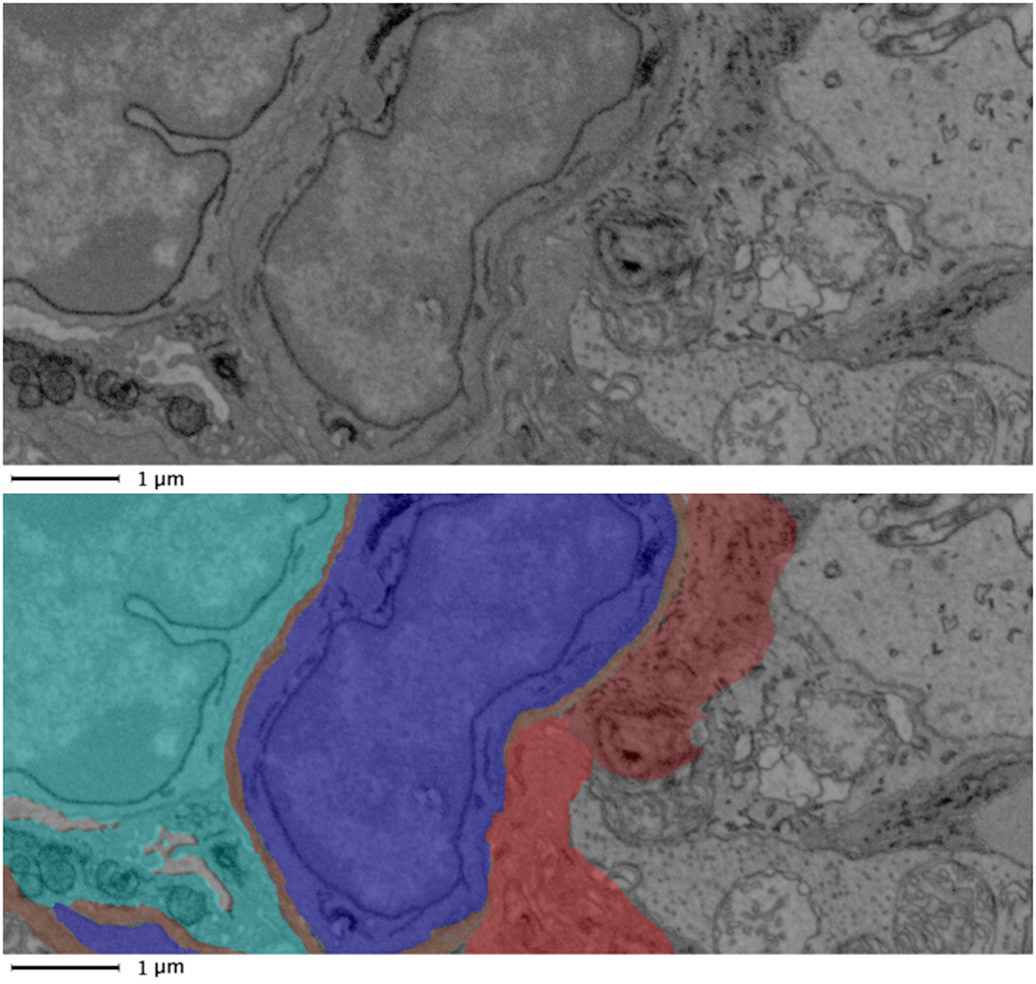
Macroglia‐pericyte direct contact. Top slide, raw data. Bottom slide, segmented features. Of the two macroglia evident in this image, represented in the bottom panel by purple and red shadings, one comes within close proximity of the pericyte (indicated in blue) by breaking through the BM (brown). An endothelial cell (aqua) is also shown.

Like the BM separating the pericytes from the endothelium, gaps were also present in the BM between the pericytes and macroglia. When such gaps occur at the outer edge of the BM, it allowed macroglia to come into direct contact with the underlying pericytes. Examples of these macroglia‐pericyte direct contacts are displayed in Figure 8 and Files S18 and S19: 10.25405/data.ncl.19087088; 10.25405/data.ncl.19087103). The closeness of the macroglia to the outer vascular BM and pericytes supports previous studies suggesting that macroglia may directly regulate retinal blood flow through paracrine signalling (Newman, 2015).

### 3.4 Neuronal cell relationship to macroglia and the vasculature

**FIGURE 9:**
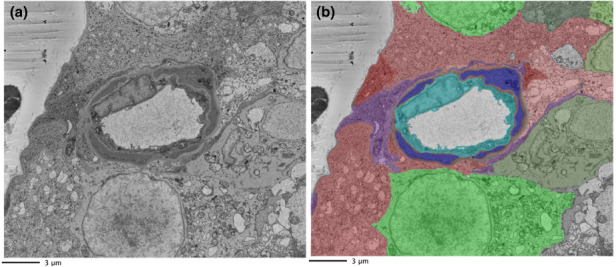
Macroglia separation of the neurons from the vasculature. a, raw and b, segmented image showing neurons (green shades) typically separated from the vasculature by surrounding macroglia (red and purple) (Endothelium: aqua; basement membrane: brown; pericytes: blue). Such features can be viewed in the animated 3D reconstruction of File S22 (10.25405/data.ncl.19086350).

**FIGURE 10:**
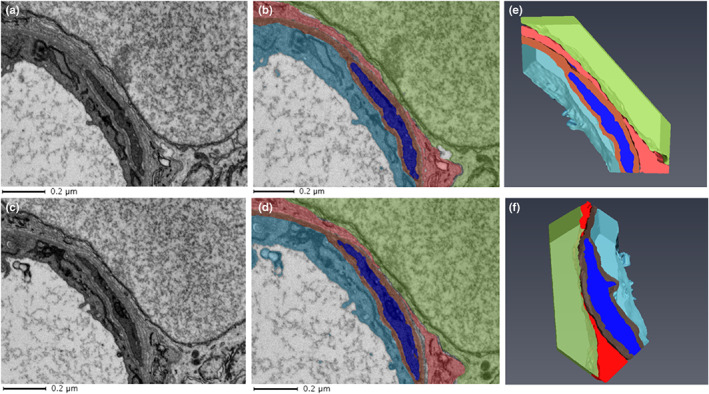
Neuron coming into close contact with vasculature. Raw (a and c) and segmented (b and d) data from consecutive sections (120 nm apart) illustrating a neuron in close proximity with the vasculature. (e and f) 3D arrangements from six consecutive sections: neurons: green shades, macroglia: red shades, endothelium: aqua; basement membrane: brown; pericytes: blue.

Although macroglia predominantly enveloped the capillaries (Figure 9), on occasions some areas of the outer capillary BM were absent from such coverage, enabling the neurons to come into contact with the BM. Examples of this are shown in Figure 10 and Files S20 and S21 (10.25405/data.ncl.19086704; 10.25405/data.ncl.19087112). This supports a previous TEM‐based study of retinal capillaries in tree shrews (Ochs et al., 2000). Such interplay between the neurons and the BM of the retinal NVU, even if infrequent, may suggest the possibility of direct neurovascular coupling; albeit this need assumes the diffusional barrier of the BM to be surmountable as, in our data, no direct contact between neurons and pericytes was observed.

Following segmentation of all features of interest the arrangements of the component cells and BM of the NVU could be visualised in 3D. An example of such reconstruction is displayed in File S22 (10.25405/data.ncl.19086350).

### 3.5 Quantitative assessment of the intercellular and cellular‐BM proximities

**FIGURE 11:**
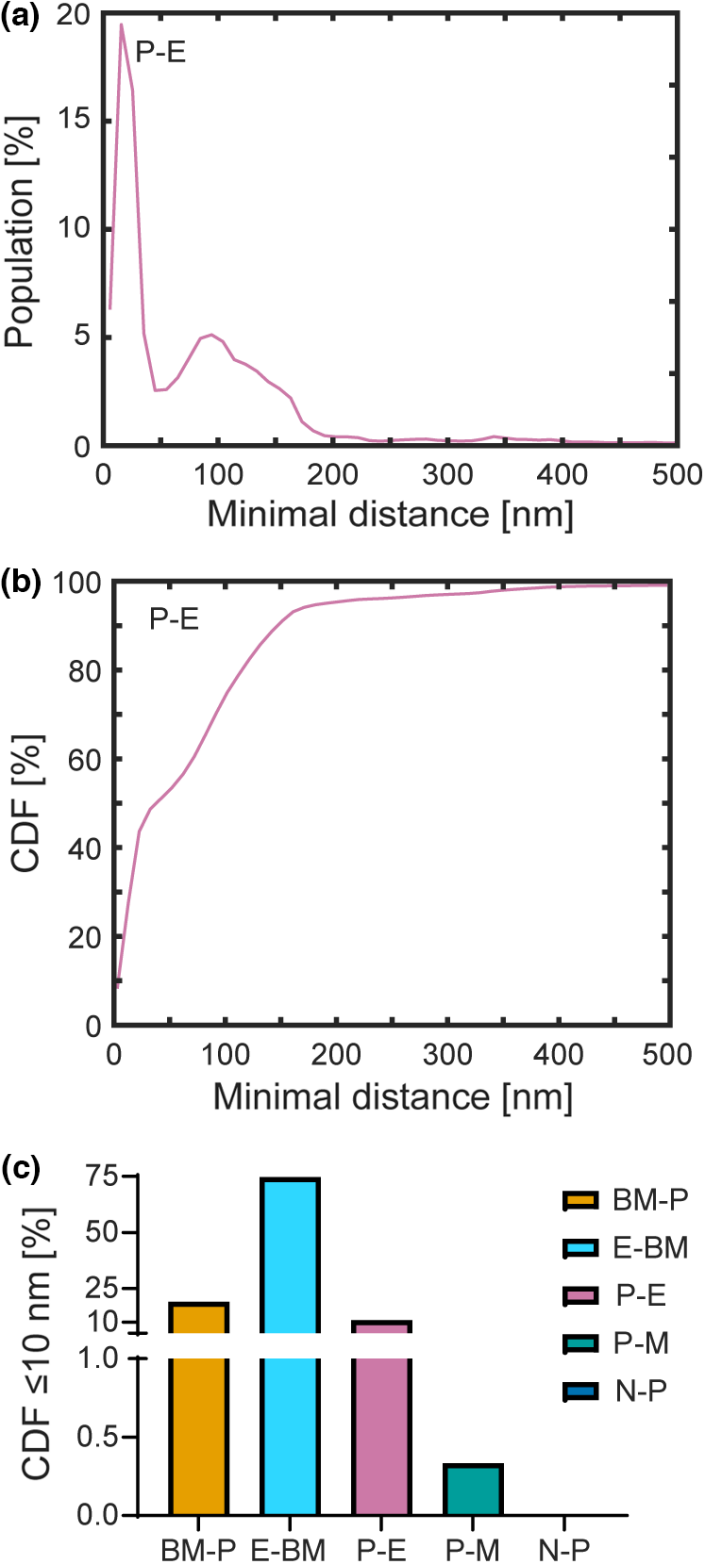
Quantitative assessment of intercellular and cellular‐BM proximities for capillary 1. (a) The stack mean population histogram of pericyte to endothelium distances. (b) The cumulative distribution function obtained from (a). (c) 10 nm readings of the cumulative distribution functions obtained from selected intercellular and cellular‐BM feature pairs to assess the degree of direct contact. BM, basement membrane; CDF, cumulative distribution function; E, endothelium; M, macroglia; N, neurons; P, pericyte.

The proximity analysis MATLAB script (File S8: 10.25405/data.ncl.19087070) enabled the determination of the minimal distances between the boundaries of the segmented features of interest. Representative results obtained from capillary 1 are shown in Figure 11. Figure 11a shows the stack mean population histogram of the percentage of pericyte boundary pixels to those of the endothelium as a function of distance. Figure 11b shows the cumulative distribution function derived from Figure 11a, enabling the total percentage of pericyte boundary pixels that lie within a given distance of the endothelium to be determined. Identical analyses were performed for other intercellular and cellular‐BM feature pairs, with a summary table of cumulative distribution function readings at 10, 100, 250, 500 and 1000 nm presented in File S23 (10.25405/data.ncl.19087133). As the pixel aspect ratio for our SBF‐SEM images is 6 nm × 6 nm, distances lying within 10 nm are essentially in direct contact. Figure 11c depicts the percentage of boundary pixels in contact for selected intercellular and cellular‐BM feature pairs. Relevant to our preceding discussions, we discovered that on average, 10.8% of pericyte boundary pixels were in direct contact with the endothelium, whereas only 0.3% were in contact with the macroglia. Consistent with our visual inspection of the images, no direct contacts were observed between the pericyte boundary pixels and those of the neurons.

### 3.6 Quantitative morphological assessment of NVU component cells and the BM

**FIGURE 12:**
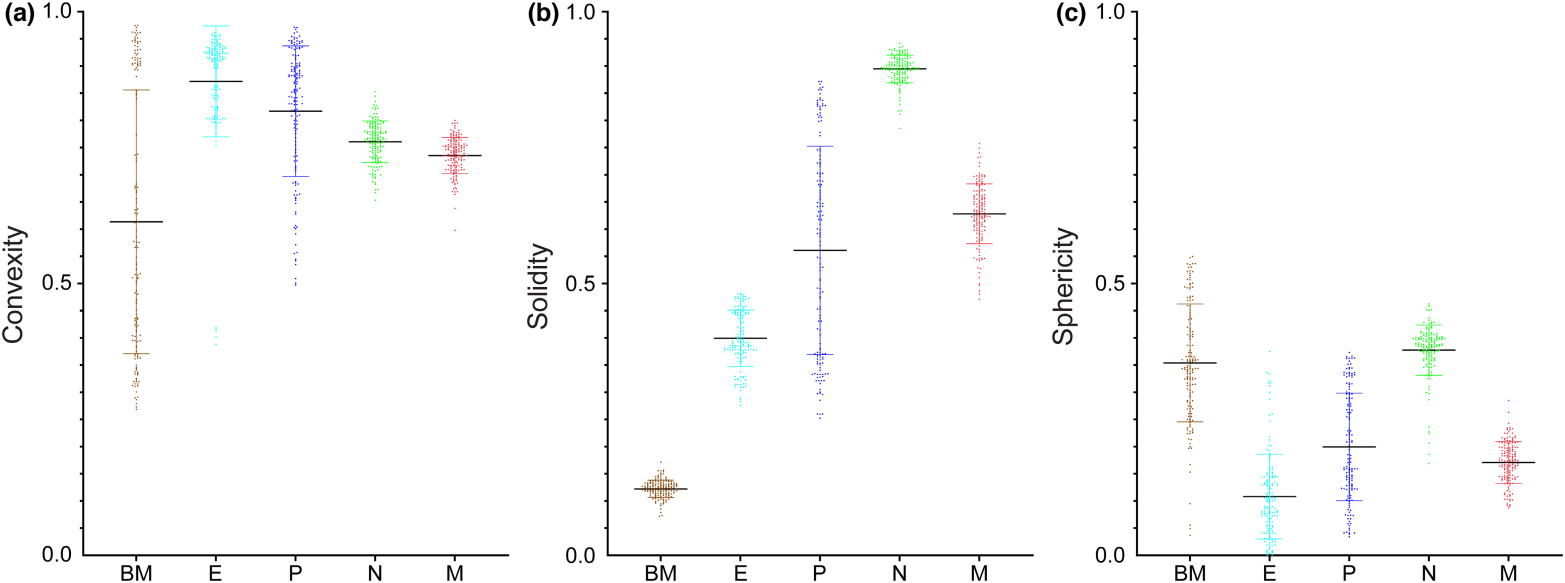
Column scatter graphs for convexity, solidity and sphericity of NVU cellular components and the BM for capillary 1. All 150 slices were analysed with each dot representing the area‐weighted mean obtained for all segmented features within each slice. Panels (a–c) convexities (convex perimeter:perimeter, CP:P), solidities (area:convex area, A:CA) and sphericities (Rmin:Rmax) of NVU component cells and the BM. Further details of calculation can be found in “Methods” section.

Another Matlab script was programmed (File S10: 10.25405/data.ncl.19087079) to quantify different morphological properties of the components of the mouse retinal NVU as described in the methods, including convexity, sphericity and solidity. Convexity and solidity were lowest for the BM when compared to the cellular components of the mouse retinal NVU (Figure 12). This was expected given the contorted, interwoven nature of the BM. Mean sphericity values were highest for neurons although this parameter showed considerable overlap for all features of the retinal NVU (Figure 12).

## 4 CONCLUSIONS

We have presented the first nanoscale examination of the anatomy of the retinal NVU (from the superficial plexus) in three spatial dimensions using SBF‐SEM. This enables visualisation and quantification in 3D of the complex architecture of the different components of the NVU which are important for regulating retinal blood flow and nutrient provision. Notable features we have revealed by examining up to 15 mm length of capillaries include: the extensive ensheathment of capillary endothelial cells and pericytes by the BM; cellular protrusions through gaps in the BM that result in regular contact of pericytes and endothelial cells, in particular, frequent occurrences of peg‐and‐socket formations; the complex patch‐work‐quilt‐like arrangement of the macroglia surrounding the vasculature; occasional regions where macroglia come into direct contact with pericytes; areas where the neuronal cells approach the capillary BM.

Together with the examination of other cellular features of the retina by SBF‐SEM (Yu et al., 2020; Meschede et al., 2021; Ratnayaka & Keeling, 2022) there is much promise for anatomically detailed, nanoscale 3D reconstructions of the entire retinal architecture to be within reach. Moreover, as advances in correlative light and electron microscopy methodologies evolve (Booth et al., 2019) there may be the opportunity to combine molecular phenotyping of particular cell types with SBF‐SEM, or allied electron microscopy, techniques.

In conclusion, this work provides new information on the heterocellular, and cellular‐BM, features of the murine retinal NVU. It thereby establishes a platform from which to explore, in future studies, qualitative and quantitative assessments of the complex heterocellular features of the retinal NVU in normal physiological settings and how these may be altered by biological (e.g. location, species) or pathological (e.g. retinopathy associated with premature birth or diabetes) circumstances.

## AUTHOR CONTRIBUTIONS

MJT, DHWS & TMC conceived and supervised the study and critically revised the manuscript; MJA, NAA & SLS performed SBF‐SEM experimentation and image analysis and reconstruction; EPT designed and performed the quantitative analysis; MJA drafted the manuscript.

## FUNDING INFORMATION

Randerson Foundation, Newcastle University. Northern Ireland Health and Social Care R&D. Division (STL/4748/13), and the Medical Research Council (MC_PC_15026).

## Supporting information


File S1
File S2
File S3
File S4.
File S5
File S6
File S7
File S8
File S9
File S10
File S11
File S12
File S13
File S14
File S15
File S16
File S17
File S18
File S19
File S20
File S21
File S22
File S23

## Data Availability

The raw data supporting this paper are available at: 10.25405/data.ncl.18545270; 10.25405/data.ncl.18550982: 10.25405/data.ncl.18551036; and 10.25405/data.ncl.18550856. Several examples of processed data and 3D visualisations are provided as Supplementary Files.
