# Supplementary material for: Investigation of heterocellular features of the mouse retinal neurovascular unit by 3D electron microscopy
Source: J Anat. 2022 Jul 16;243(2):245–57. doi: 10.1111/joa.13721 (PMC10335381; doi:10.1111/joa.13721)
Supplement: Supplementary file 1 — File S1 File S2 File S3 File S4. File S5 File S6 File S7 File S8 File S9 File S10 File S11 File S12 File S13 File S14 File S15 File S16 File S17 File S18 File S19 File S20 File S21 File S22 File S23 [file JOA-243-245-s001.docx]

**Supplementary File 1 – Raw data for capillary 1. Animated view of** 150 serial sections of retinal mouse capillary 1 covering a depth of 18μm.

**Supplementary File 2 – Raw data for capillary 2. Animated view of** 150 serial sections of retinal mouse capillary 2 covering a depth of 15μm.

**Supplementary File 3 – Raw data for capillary 3. Animated view of** 150 serial sections of retinal mouse capillary 3 covering a depth of 18μm.

**Supplementary File 4 – Raw data for capillary 4. Animated view of** 100 serial sections of retinal mouse capillary 4 covering a depth of 12μm.

**Supplementary File 5: Identification of cells from plasma membrane outlines.** The cellular components of capillaries and the neurovascular units were each outlined by carefully drawing along the cellular membranes identified by digital zooming of the raw data images. The plasma membranes bordering neighbouring cells are indicated in this image by white arrows.

**Supplementary File 6: Characterising astrocyte by presence of cell nucleus.** When assessing a macroglial cell through a stack of 150 images there were several sections in which a nucleus was evident. This is indicative of an astrocyte (see Methods 2.3) and, therefore, this cell was segmented in purple to distinguish it from other macroglial cells.

**Supplementary File 7: Semi-automated detection of pericyte-endothelial interactions.** Capillaries were analysed under the Arivis analysis pipeline (see Methods) which semi-automatically detected endothelial-pericyte closeness and depicted such occasions in green highlights. Data shown from capillary 1 from which the pipeline assessed all 150 sections. One section (corresponding to section 36) is shown in panels a-d. a) segmented features are endothelium: aqua, basement membrane: brown, pericyte: blue. (b)-(d) close contacts are displayed with green elements as quantified in Arivis with user-instructed minimal distances of (b) 5 pixels, (c) 3 pixels and (d) 1 pixel.

**Supplementary File 8 – MATLAB proximity analysis code.**

**Supplementary File 9 – Pictographic summary of morphological analysis features.** A schematic of the morphological assessments described in the methods.

**Supplementary File 10 - MATLAB morphological analysis code.**

**Supplementary File 11 – 3D model for capillary 1 vasculature.** 3D model of mouse capillary 1 vasculature. Vascular components follow as blue: pericyte, aqua: endothelium; and brown: basement membrane.

**Supplementary File 12 – 3D model for capillary 2 vasculature.** 3D model of mouse capillary 2 vasculature. Pericyte: blue, endothelium: aqua, basement membrane: brown.

**Supplementary File 13 – 3D model for capillary 3 vasculature.** 3D model of mouse capillary 3 vasculature. Pericyte: blue, endothelium: aqua, basement membrane: brown.

**Supplementary File 14 – Additional example of peg-and-socket formation spanning across multiple sections.** An example of peg-and-socket formation in addition to those of Figures 5-6. Pericyte: blue, endothelium: aqua, basement membrane: brown.

**Supplementary File 15 – Animated 3D model of peg-and-socket example 1.** 3D model of peg and socket formation. Pericyte: blue, endothelium: aqua, basement membrane: brown.

**Supplementary File 16 – Animated 3D model of peg-and-socket example 2.** Additional example of a 3D model of peg and socket formation. Pericyte royal blue; endothelium: aqua; basement membrane brown.

**Supplementary File 17 - Macroglial cell wrapping 3D model.** 3D model of macroglial cell wrapping around a vessel (for ease of visualisation of macroglia, the vascular cells have been removed from the video). Macroglia cells assigned shades of red. One cell identified as an astrocyte is indicated in a purple shade.

**Supplementary File 18 - Macroglia cell closeness to pericytes example 2.** An additional example of macroglia cell closeness to pericytes in consecutive sections (120 nm apart).  **a**, **c** raw data. **b**, **d** segmented data. Macroglia cells assigned shades of red. One cell identified as an astrocyte is indicated in a purple shade, pericytes in blue, endothelium in aqua and basement membrane in brown.

**Supplementary File 19 - Macroglia cell closeness to pericytes example 3. Another** example of macroglia cell closeness to pericytes from the same specimen as in Supplementary File 15. **a**, **c** raw data. **b**, **d** segmented data. Macroglia cells assigned shades of red. One cell identified as an astrocyte is indicated in a purple shade, pericytes in blue, endothelium in aqua and basement membrane in brown.

**Supplementary File 20 – 3D model of neuron coming in to close contact with vasculature.** 3D model of neuronal cell closeness to pericytes. Neurons were assigned shades of green, pericyte: blue, endothelium: aqua and basement membrane: brown.

**Supplementary File 21- Neuron coming into close contact with vasculature example 2.** An additional example of neuronal cell closeness to basement membrane displayed in consecutive images (120 nm apart). **a**, **c** raw data. **b**, **d** segmented data. Neurons were assigned shades of green, pericyte: blue, endothelium: aqua, basement membrane: brown.

**Supplementary File 22 – 3D model of neurovascular unit.** A 3D model of all components of the neurovascular unit along 18 μm length of capillary 1 is displayed. Pericyte: blue, endothelium: aqua, basement membrane: brown. Remaining cellular components are: neurons, green; macroglia, red shades and one cell identified as an astrocyte is assigned a purple shade.

**Supplementary File 23 – Summary of the proximity analyses for the selected NVU feature pairs.** Means and standard deviations of the cumulative distribution functions at increasing distances up to 1000nm are provided for the intercellular and cellular-BM pairs featured in Figure 11C.
